# MULTIMODAL PERSONALIZED CHRONOTHERAPY IMPROVES SLEEP IN ADULTS WITH MILD COGNITIVE IMPAIRMENT: A RANDOMIZED TRIAL

**DOI:** 10.1093/geroni/igz038.1342

**Published:** 2019-11-08

**Authors:** Ryan S Falck, John R Best, Jennifer C Davis, Patrick Chan, Daniel Backhouse, Glenn J Landry, Teresa Liu-Ambrose

**Affiliations:** 1 University of British Columbia, Vancouver, British Columbia, Canada; 2 University of British Columbia - Okanagan Campus, Kelowna, British Columbia, Canada

## Abstract

Poor sleep is common among older adults with Mild Cognitive Impairment (MCI) and may contribute to their increased risk for dementia. Chronotherapy is a set of intervention strategies which can improve sleep quality by strengthening the entrainment of the biological clock to the solar light-dark cycle, and includes strategies such as: 1) bright light therapy (BLT); 2) physical activity (PA); and 3) good sleep hygiene. Thus, in this 24-week randomized controlled trial (RCT; NCT02926157), we aimed to examine the efficacy of a multimodal, personalized chronotherapy intervention to improve sleep quality among older adults with MCI. Ninety-six older adults (65+ years) with MCI were randomized to either: 1) a multimodal personalized chronotherapy group (INT); or 2) a waitlist-plus-education control group (CON). Participants allocated to the INT received four once-weekly, general sleep hygiene education classes, followed by 20 weeks of 1) individually-timed BLT; and 2) bi-weekly, individually-tailored PA counselling in conjunction with receiving a consumer-available PA tracker (Fitbit® FlexTM). We found a significant group x time interaction for objectively measured sleep fragmentation (5.01; p< 0.01) and also for Pittsburgh Sleep Quality Index (PSQI) score (p= 0.03), such that the INT: 1) maintained sleep fragmentation while CON worsened at 12 weeks (p< 0.01); and 2) had improved PSQI score compared to CON at both 12 weeks (p< 0.01) and 24 weeks (p= 0.04). Our results provide novel evidence that a multimodal personalized chronotherapy approach may promote both objective and subjective aspects of sleep quality in older adults with MCI.

